# A formulated poly (I:C)/CCL21 as an effective mucosal adjuvant for gamma-irradiated influenza vaccine

**DOI:** 10.1186/s12985-021-01672-3

**Published:** 2021-10-09

**Authors:** Ailar Sabbaghi, Masoud Malek, Sara Abdolahi, Seyed Mohammad Miri, Leila Alizadeh, Mehdi Samadi, Seyed Reza Mohebbi, Amir Ghaemi

**Affiliations:** 1 Department of Influenza and Other Respiratory Viruses, Pasteur Institute of Iran, P.O.Box: 1316943551, Tehran, Iran; 2 Department of Microbiology, Science and Research Branch, Islamic Azad University, Tehran, Iran; 3 Shefa Neuroscience Research Center, Khatam Alanbia Hospital, Tehran, Iran; 4 Department of Medical Virology, Tehran University of Medical Sciences, Tehran, Iran; 5 Gastroenterology and Liver Diseases Research Center, Research Institute for Gastroenterology and Liver Diseases, Shahid Beheshti University of Medical Sciences, Tehran, Iran

**Keywords:** Gamma-irradiated influenza vaccine, Adjuvants, Protective immunity, CCL21, Poly (I:C)

## Abstract

**Background:**

Several studies on gamma-irradiated influenza A virus (γ-Flu) have revealed its superior efficacy for inducing homologous and heterologous virus-specific immunity. However, many inactivated vaccines, notably in nasal delivery, require adjuvants to increase the quality and magnitude of vaccine responses.

**Methods:**

To illustrate the impacts of co-administration of the gamma-irradiated H1N1 vaccine with poly (I:C) and recombinant murine CCL21, either alone or in combination with each other, as adjuvants on the vaccine potency, mice were inoculated intranasally 3 times at one-week interval with γ-Flu *alone or* with any of the three adjuvant combinations and then challenged with a high lethal dose (10 LD50) of A/PR/8/34 (H1N1) influenza virus. Virus-specific humoral, mucosal, and cell-mediated immunity, as well as cytokine profiles in the spleen (IFN-γ, IL-12, and IL-4), and in the lung homogenates (IL-6 and IL-10) were measured by ELISA. The proliferative response of restimulated splenocytes was also determined by MTT assay.

**Results:**

The findings showed that the co-delivery of the γ-Flu vaccine and CCL21 or Poly (I:C) significantly increased the vaccine immunogenicity compared to the non-adjuvanted vaccine, associated with more potent protection following challenge infection. However, the mice given a combination of CCL21 with poly (I:C) had strong antibody- and cell-mediated immunity, which were considerably higher than responses of mice receiving the γ-Flu vaccine with each adjuvant separately. This combination also reduced inflammatory mediator levels (notably IL-10) in lung homogenate samples.

**Conclusions:**

The results indicate that adjuvantation with the CCL21 and poly (I:C) can successfully induce vigorous vaccine-mediated protection, suggesting a robust propensity for CCL21 plus poly (I:C) as a potent mucosal adjuvant.

## Introduction

Appropriate inactivation techniques, which abolish viral infectious titers while preserving the immunogenicity of viruses, should be employed to develop whole-virion vaccines able to induce cross-immunity. Inducing chemical changes using formaldehyde or β-propiolactone along with incorporation of physical treatments such as ultraviolet light or gamma-rays are the prevailing procedures used for the preparation of inactivated influenza viruses [1]. However, the comparison of three different sterilization methods has shown that gamma-irradiated influenza A virus (γ-Flu) significantly contributes to T-cell cross-reactivity, due to the ability of gamma-rays to preserve most of the antigenic structure and biological integrity of proteins during treatment, making it a promising vaccine candidate to overcome the low efficacy of current Flu-vaccines against antigenic variants of influenza virus [1–6].

More importantly, radiation conditions including gamma-irradiation dose and the temperature of radiation, -as well as the Bremsstrahlung process, the secondary radiation produced during treatment with gamma-irradiation, may introduce unwanted structural damages in viral proteins upon the pathogen inactivation [6, 7]. Recently, adverse effects of non-optimized experimental inactivation conditions, such as high radiation dose and high temperature, on the immunogenicity of the γ-Flu vaccine have been evaluated [6]. Besides, mucosal immunization by intranasal administration of inactivated vaccines without appropriate mucosal adjuvants is often not sufficiently effective [8–10]. Therefore, regarding nasal gamma-based vaccine development, the use of proper adjuvants may provide a rational approach for increasing vaccine efficacy [9, 11]. Of note, vaccine adjuvant incorporation strategy may also be beneficial for decreasing the reactogenicity of whole inactivated Flu viruses by reducing vaccine doses required to achieve sufficient protective immunity [11, 12].

Targeting dendritic cell (DC) receptors by antigens such as polyriboinosinic-polyribocytidylic acid (poly (I:C)) and chemokine (C–C motif) ligand 21 (CCL21) has been shown to significantly improve vaccine immunization via supporting adaptive immunity, indicating their potential clinical value [10, 13–17]. Poly (I:C) is an artificial form of viral double-stranded RNA (dsRNA), which induces the activation of toll-like receptor 3 (TLR3) and cytoplasmic dsRNA sensors (such as melanocyte differentiation-associated 5 (MDA5), retinoic acid-inducible gene I (RIG-I), and the NLR *pyrin domain* containing *3* (*Nlrp3*)), as well as the dictation of their downstream signaling pathways, resulting in cytokine production, up-regulation of co-stimulatory molecules and DCs maturation [18]. Mechanistically, the adjuvant activity of poly (I:C) is more related to its superior ability for inducing type 1 interferon (IFN-1), which is directly associated with the linkage of innate and adaptive immune responses [13]. CCL21, a ligand for CC chemokine receptor 7 (CCR7), has also been known to be vital for priming responses of adaptive immunity. Owing to the role of CCL21 as the main regulator of lymphatic migration of CCR7^+^ immune cells, notably DCs and T-helper 1 (Th1) lymphocytes to T-cell regions of secondary lymphoid organs, which is a critical step in orchestrating adaptive T-cell responses, it can be considered as an attractive adjuvant to facilitate vaccines to boost immunity [15, 19, 20]. In addition, the pre-clinical and clinical researches using different tumor models have revealed the efficacy of poly (I:C) and CCL21 for using as a cancer vaccine adjuvant, making them promising candidates for developing a reasonable and safe vaccine adjuvant incorporation strategy [15, 21, 22].

Recently, the activity of poly (I:C) as a mucosal vaccine adjuvant and adaptive-immunity activator has been successfully assessed in animal models of influenza infection [8, 10, 23–25]. CCL21 is also the well-studied lymphoid chemokine adjuvant, and when applied in a mucosal prime/boost regimen against herpes simplex virus type-1 (HSV-1), led to further improvement of protection by the vaccine [26]. Accordingly, this study was undertaken to evaluate whether intranasal immunization of BALB/c mice with gamma-irradiated H1N1 vaccine plus poly (I:C) and recombinant murine CCL21, as adjuvants, induced improved protective immune responses against lethal influenza virus challenge.

## Materials and methods

### Cells and viruses

The mouse-adapted A/H1N1/Puerto Rico/8/1934 (PR8) was procured from the Pasteur Institute (Tehran, Iran), grown by infecting Madin-Darby Canine Kidney (MDCK) monolayer cell cultures at a multiplicity of infection (MOI) of 6, and incubated at 37 °C in a humidified atmosphere with 5% CO_2_. When the complete cytopathic effect (CPE) was observed, virion-containing culture supernatants were harvested, pooled, clarified by centrifugation (4 °C, 3000 rpm, and 15 min), concentrated by ultrafiltration system with 100 kDa cut-off, and then tittered in MDCK monolayers using the median tissue culture infectious dose (TCID50) assay. Upon intranasal administration of mice with 50 μl of the infectious virus, the median lethal dose (LD50) of PR8 for the viral challenge was also determined. Both TCID50 and LD50 titers were measured using the method of Spearman-Karber [27].

### Vaccine preparation

The γ-Flu vaccine was prepared according to a previously described method [11]. Briefly, concentrated PR8 stocks were kept frozen on dry ice upon treatment and received 28 kGy of gamma-irradiation from a ^60^Co γ-ray source (GammaCell 220; MDS *Nordion*, Ottawa, Canada), followed by the evaluation of the sterility using the safety test. Lack of visible CPE upon consecutive passages illustrated the complete loss of virus infectivity**.** Furthermore, Hemagglutination assay (HA) and Sodium dodecylsulfate-polyacrylamide gel electrophoresis (SDS-PAGE) were respectively used to confirm the structural integrity of the hemagglutinin and the quality of viral proteins following exposure to 28 kGy.

### Preparation of adjuvants

Recombinant murine CCL21 was obtained from R&D Systems (Minneapolis, MN, USA) and delivered in 50 μl of sterile PBS with 0.05% normal mouse serum (Sigma) as per the manufacturer's instructions. Poly (I:C) powder was also purchased (InvivoGen, San Diego, USA) and dissolved in sterile PBS at a final concentration of 1 μg/ml following the manufacturer's instructions. Aliquots were stored frozen until assayed.

### Mice immunization and viral challenge

**Fig. 1:**
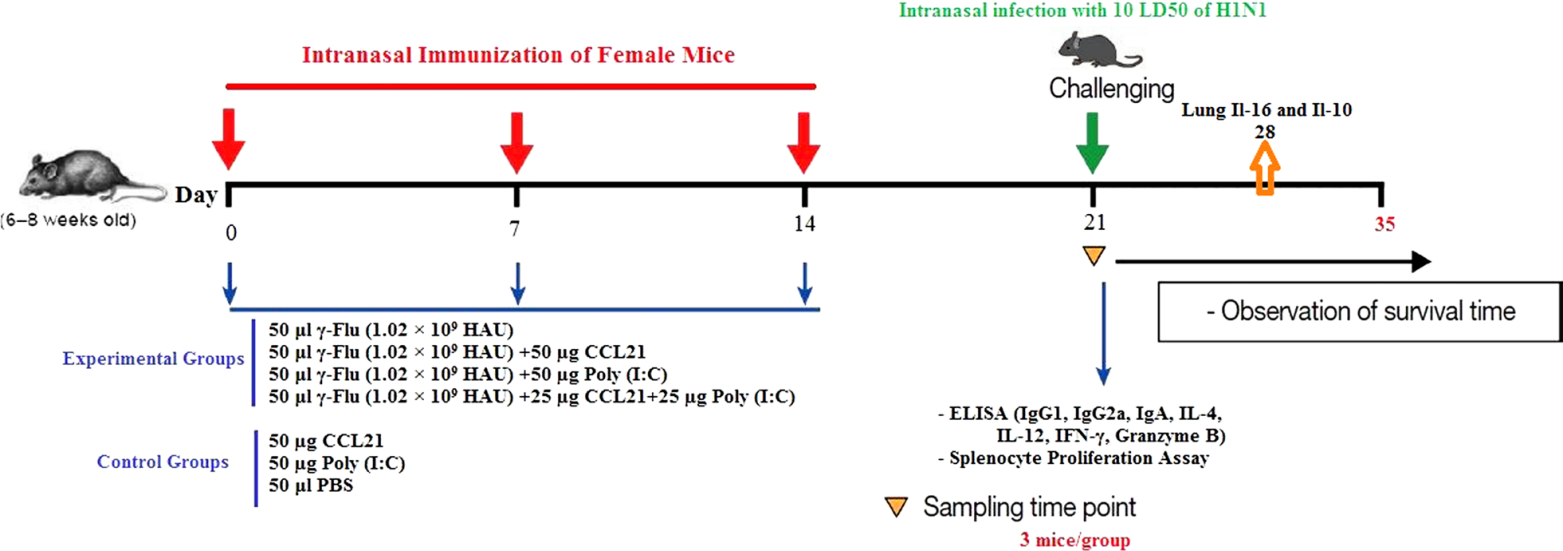
Mouse immunization schedule

Female BALB/c mice (6–8 weeks old) supplied by Pasteur Institute (Karaj, Iran) were housed under specific-pathogen-free conditions and randomly assigned to seven groups (15 mice in each group). The experimental groups were anesthetized intraperitoneally with the ketamine-xylazine solution at the dose of 50 mg/kg and 20 mg/kg, respectively, and intranasally immunized on days 0, 7, and 14 with either 50 μl per mouse of γ-Flu (1.02 × 10^9^ HAU) with or without each adjuvant separately or with the combination of both adjuvants at a final concentration of 50 µg per mouse. Furthermore, to construct control groups, anesthetized control mice were treated with 50 µg Poly (I:C), 50 µg CCL21, or 50 μl PBS as well (Fig. 1). Of note, the γ-Flu vaccine antigen content and subsequently vaccine doses are measured according to those reported by David et al. [6].

One week post-final immunization, mice were anesthetized, challenged intranasally with 10 LD50 of A/PR/8/34 (H1N1) influenza viruses, and monitored daily for a period of 14 days for signs of morbidity and mortality. A weight loss exceeding 25% was considered as the experimental endpoint, and mice reaching this endpoint were culled.

Determination of the virus titer using MDCK/Hemagglutination assay (HA) was applied on day 4 after viral challenge. For the assay, the lungs homogenates (three mice in each group) were frozen and thawed, and their supernatants diluted from 10- to 10^6^-fold (in tenfold steps), were added into triplicate wells seeding MDCK cells. After incubation, the titer of virus was measured by co-incubation of the MDCK supernatant with chicken red blood cells. Lung virus titers were analyzed by interpolation of the dilution endpoint that infected 50% of wells, and then expressed as log_10_ TCID50.

### Systemic antibody-mediated immune responses

At one week after the final immunization, serum was obtained by retro-orbital bleeding from individual mice of each group (n = 3), and the level of influenza virus-specific total IgG and IgG isotypes in the sera were determined by enzyme-linked immunosorbent assay (ELISA), as previously described [11]. Briefly, wells of 96-microwell ELISA plates (Nunc, Denmark) were coated with 4 μg/ml of the γ- PR8 and then blocked with blocking buffer (PBS with 5% BSA, 0.1% Tween 20) for 2 h at room temperature (RT). Following incubation, two-fold serially diluted samples were applied to each well and incubated at 37 °C for 2 h. Secondary goat anti-mouse IgG, IgG1, and IgG2a horseradish peroxidase (HRP)-conjugated antibodies (Sigma-Aldrich) were then loaded to each well and incubated for 2 h at RT. Plates were developed by adding tetramethylbenzidine (TMB, Sigma-Aldrich) as HRP substrate to appropriate wells in the dark for 30 min. After halting the reaction with 2 M H_2_SO_4_, the optical density (OD) at 450 nm was measured using an automated ELISA plate reader (Multiskan FC, Thermo Scientific, Waltham, MA, USA). The endpoint ELISA titers were expressed as the serum dilution created the strongest signal compared to the baseline (at least 3 times).

### Influenza-specific secretory IgA responses

Mucosal antibody responses to vaccination were evaluated by ELISA assay on day 21. To obtain nasal and Bronchoalveolar Lavage (BAL) fluids, three mice per group were randomly chosen and sacrificed intraperitoneally with an over dose of ketamine-xylazine solution, followed by washing the nasal cavities and flushing the lungs with 1 ml PBS containing the Roche's complete protease inhibitor cocktail for 5 times. Cellular debris was removed by centrifugation (400×*g* for 10 min at 4 °C) and supernatants were collected and then subjected to antibody analysis by ELISA assay using Sigmaˈs HRP goat anti-mouse (IgA) secondary antibody as described above.

### Splenocytes proliferation assay

To analyze cell-mediated immune responses, one week after the third immunization, the splenocytes were harvested from the spleen of three mice per group, cultured in triplicate in 96-well plates at a density of 2 × 10^5^ cells per well, and then incubated at 37 °C for 48 h under a humidified atmosphere of 5% CO_2_ and 95% air atmosphere with γ-PR8 (4 μg/ml) as the specific antigen matched with the vaccine groups, or without stimuli (medium only) as the negative control. Finally, cell proliferation assay was conducted by the addition of MTT (3-(4,5-dimethylthiazoyl)-2,5diphenyltetrazolium bromide) solution to appropriate wells, followed by the addition of dimethyl sulfoxide to dissolve formazan crystals produced upon MTT reduction via the mitochondrial activity of living cells. Absorbance values were then measured at 540 nm using the ELISA plate reader. The stimulating index (SI) [28] was calculated as follows:

SI = (OD of stimulated cells-OD of unstimulated cells)/OD of unstimulated cells [11].

### Spleen cell cytokine responses

On day 21, the concentration of Th1 (IFN-γ, IL-12) and Th2 (IL-4) cytokines present in the supernatants of splenocytes from control and test animals were evaluated using commercially available cytokine-specific ELISA kits (R&D, USA) as per the manufacturer's instructions. For this, the mononuclear cells were isolated and stimulated as mentioned above. Forty-eight to 72 h post-stimulation, the supernatants were collected and then assayed for the presence of respective cytokines using a quantitative sandwich ELISA assay. Finally, the plates were read at 450 nm and values were expressed as optical densities. The cytokine level of each sample was then determined by interpolation of OD on a standard curve created with known quantities of the cytokine. For each mouse, all tests were performed in triplicate.

### Analysis of granzyme B activity

To investigate the influenza-specific cytolytic activity, 7 days after the last administration, the amount of granzyme B *(GrB)* protein, as a marker of activated cytotoxic T cells, in the supernatants of γ-PR8-stimulated mononuclear cells from spleens of three mice in each group was measured according to the commercially available granzyme B sandwich-based ELISA kit (R&D, USA) instructions. Samples and standards were evaluated at an optical density of 450 nm. All tests were carried out in triplicate for each mouse.

### Analysis of lung cytokines

To study inflammatory cytokine profile, a week after the viral challenge, the lung tissues from three mice per group were collected, homogenized, and centrifuged at 9000×*g* for 10 min [29]. Supernatants were then subjected to cytokine analysis by a quantitative sandwich ELISA assay using commercially available ELISA kits (R&D, USA) according to protocols recommended by the manufacturers. All specimens were analyzed in triplicate.

### Statistical analysis

Data were expressed as means ± SD. Results between the different groups were compared by one-way ANOVA test. A Kaplan–Meier curve was also used for the analysis of survival rates. The statistical significance level was set at P value ≤ 0.05. The statistical software SPSS version 16.0 (SPSS Inc., Chicago, IL, USA) was used for statistical analysis.

## Results

### CCL21-Poly (I:C) adjuvanted vaccine improved systemic antibodies and cell-mediated immunity in mice

**Fig. 2:**
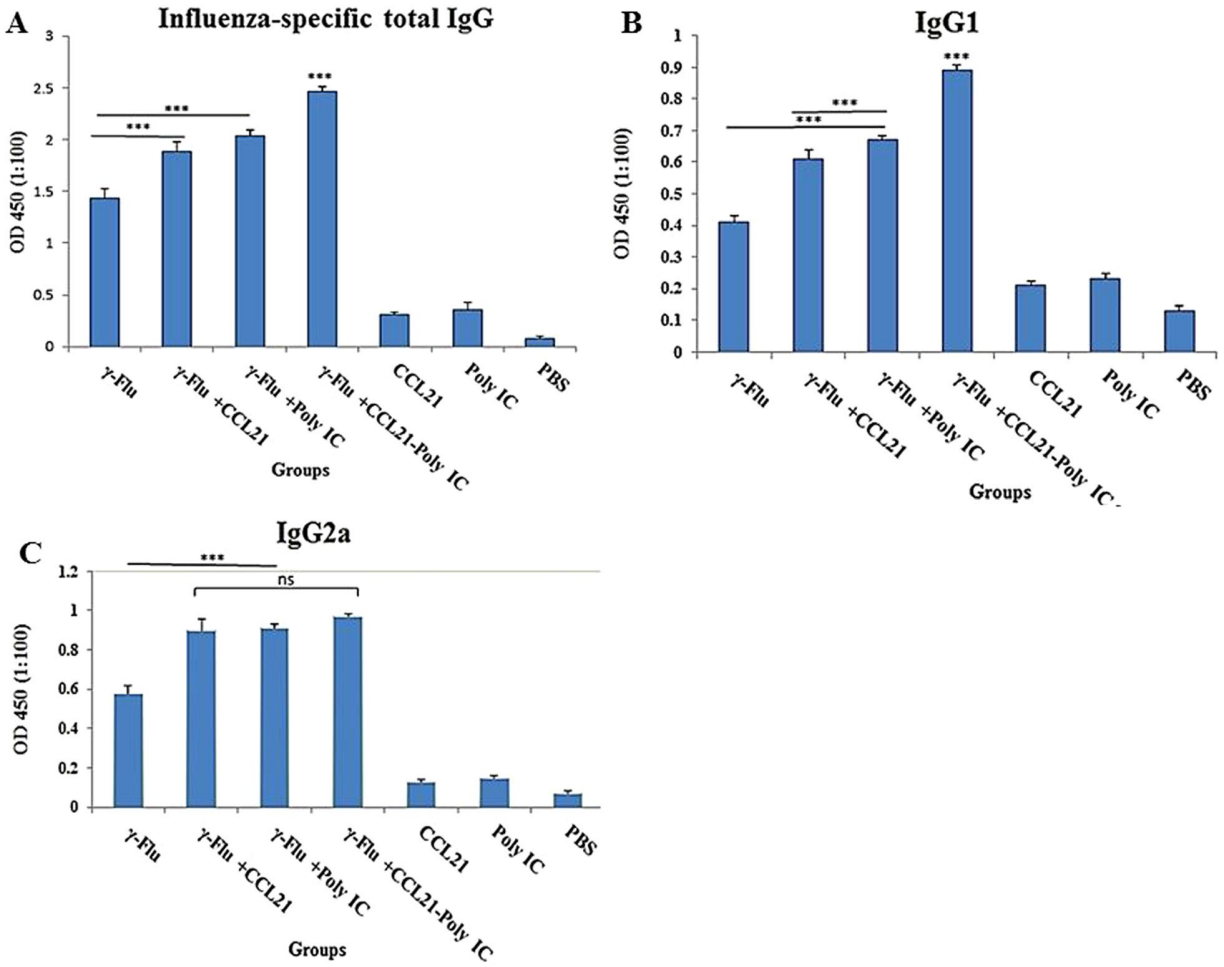
Effects of CCL21 and Poly (I:C) as adjuvants on specific IgG antibody levels. Columns represent relative antibody expression in each treated group (n = 3). The measured values for influenza-specific total IgG (**a**), IgG1 (**b**), and IgG2a (**c**) in immune sera were reported as the mean ± SD, and One way ANOVA is used to calculate the statistical significance. Differences between all treatment groups and control groups were statistically high significant (***p < 0.001). The highest level of total IgG and IgG1 isotype was also observed in mice primed with γ-Flu plus CCL21-Poly (I:C) while there was no significant difference among the groups immunized with γ-Flu-CCL21, γ-Flu-Poly (I:C), or γ-Flu-CCL21-Poly (I:C) in the number of IgG2a secreting cells

*To characterize the mucosal adjuvant efficacy of murine recombinant CCL21 and Poly (I:C) for* γ*-inactivated influenza A (subtype H1N1) vaccine, immunoglobulin G antibodies were examined in the sera collected from mice* inoculated intranasally 3 times at one-week interval with γ-Flu, γ-Flu*-CCL21,* γ-Flu*-Poly (I:C), or* γ-Flu*-CCL21-Poly (I:C). Our data illustrated the significant enhancement of influenza-specific total IgG titer in mice immunized* with either γ-Flu alone or with any of the three adjuvant combinations compared to mice treated with *CCL21, Poly (I:C), and PBS* (p < 0.001)*. As shown in *Fig. 2a*, total IgG responses in mice given the* γ-Flu with each adjuvant separately or with the combination of both adjuvants *were considerably higher than those received* non-adjuvanted vaccine (p < 0.001)*. Of note, co-delivery of* CCL21 and Poly (I:C) as adjuvant *afforded significantly higher levels of total immunoglobulin titer than* any adjuvant alone (p < 0.001)*.*

To evaluate the induced adaptive immune responses, sera were collected *on day 7 following the third immunization* and then subjected to IgG subclasses analysis *using* an endpoint Ab enzyme-linked immunosorbent assay*. In comparison with* the γ-Flu or control groups, *intranasal immunization with* γ-Flu *adjuvanted by CCL21, Poly (I:C), or CCL21-Poly (I:C) elicited considerably higher levels of IgG isotypes* (Fig. 2a) (p < 0.001)*. Importantly, the* CCL21 and Poly (I:C) combination adjuvant *significantly* enhanced the *serum levels of the Th2-associated IgG1* compared to the other two adjuvants (p < 0.001) while *the CCL21 adjuvant group, Poly (I:C) adjuvant group, and CCL21-Poly (I:C) adjuvant group responded with similar levels of the IgG2a* (Fig. 2b, c). However, *adjuvant*ed platforms induced a high level of IgG2a, compared with IgG1, indicating that vaccine adjuvant formulation promoted an enhanced balanced Th1/Th2 immune response. *There was no significant difference* in the levels of total IgG, IgG1, and IgG2a between control groups*.*

### Co-delivery of CCL21 and Poly (I:C) as adjuvant considerably improved the mucosal antibody responses in BALF samples

**Fig. 3:**
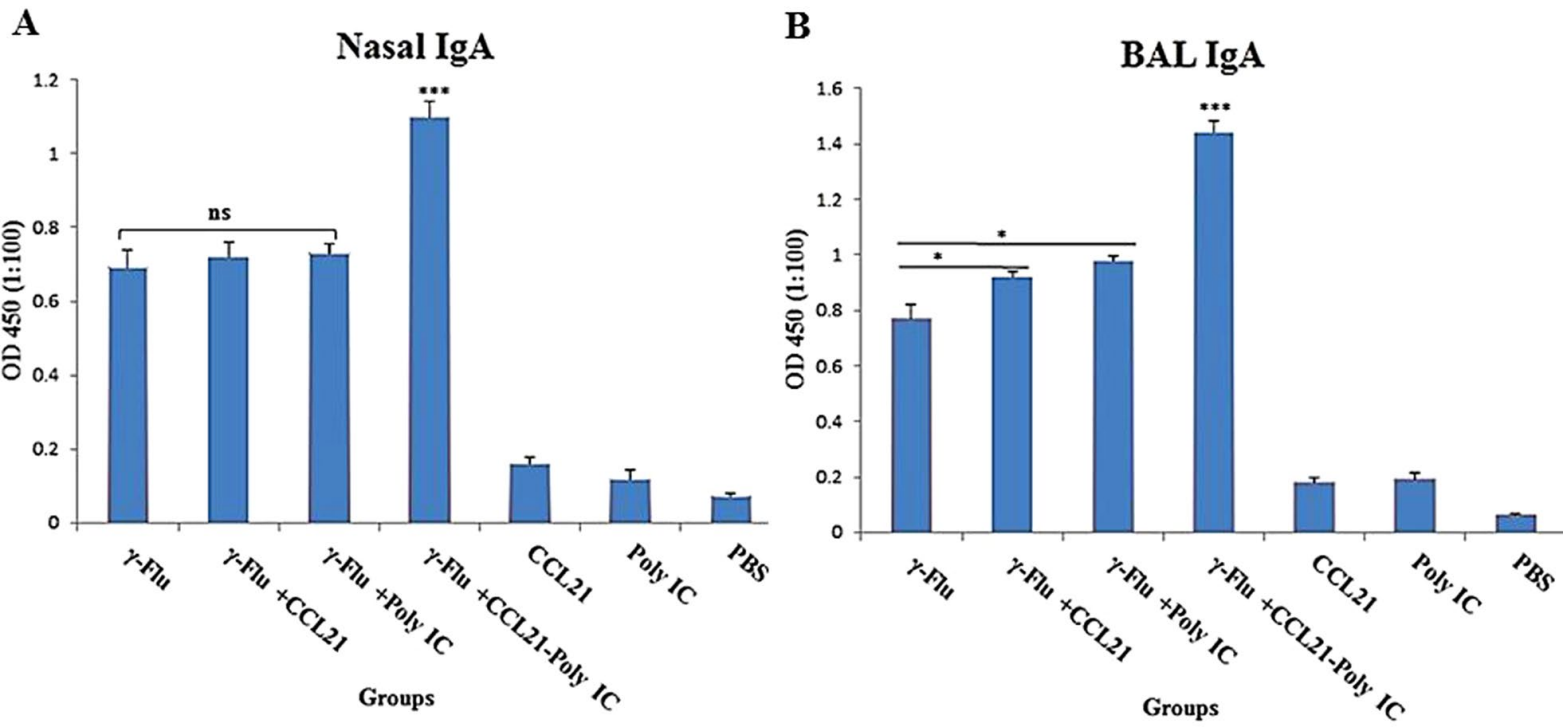
Analysis of antibody response in nasal (**a**) washes and BAL (**b**) fluid of immunized mice using the ELISA method. Data are expressed as means ± SD of three mice per group and difference among individual groups is determined by One way ANOVA and shown as *** for p < 0.001, * for p < 0.05, and ns for a nonstatistical difference. Compared to different adjuvant formulations or control groups, CCL21-Poly (I:C) significantly induced nasal and BALF IgA response of the γ-Flu vaccine (p < 0.001)

To further evaluated the potential applicability *of CCL21 and Poly (I:C)* as mucosal adjuvants, nasal washes and BAL fluid of immunized mice were analyzed for IgA antibodies using the ELISA method on day 21. In the comparison between vaccinated and control groups, a statistically significant difference (p < 0.001) in the IgA profiles were recorded (Fig. 3). *The* results also illustrated that mice immunized with vaccine plus *CCL21-Poly (I:C)* developed significantly higher influenza-specific IgA titers in BALF samples compared to the other adjuvant or non-adjuvant groups (p < 0.001) (Fig. 3b). A similar pattern was observed for the IgA antibody in the nose of mice receiving γ-Flu*-CCL21-Poly (I:C)* in comparison to those given γ-Flu, γ-Flu*-CCL21*, and γ-Flu*-Poly (I:C)* (p < 0.001) (Fig. 3a)*,* indicating a synergistic effect *of CCL21 and Poly (I:C) on the induction of mucosal antibody-mediated immunity*. However, nasal IgA response rates did not differ significantly between the groups that received γ-Flu with or without each adjuvant separately, whereas the mice in the γ-Flu*-CCL21 and* γ-Flu*-Poly (I:C)* groups displayed higher levels of BALF IgA than those given γ-Flu alone (p < 0.05). This indicates that *any* adjuvant alone improved the mucosal antibody immunity of the inactivated vaccine in BAL fluids while there was no effect on the vaccine-induced nasal IgA response. The statistical analysis of mucosal IgA antibody titers did not reveal a significant difference among control groups.

### The proliferative response of restimulated splenocytes was significantly enhanced by the CCL21-Poly (I:C) combination adjuvant

**Fig. 4:**
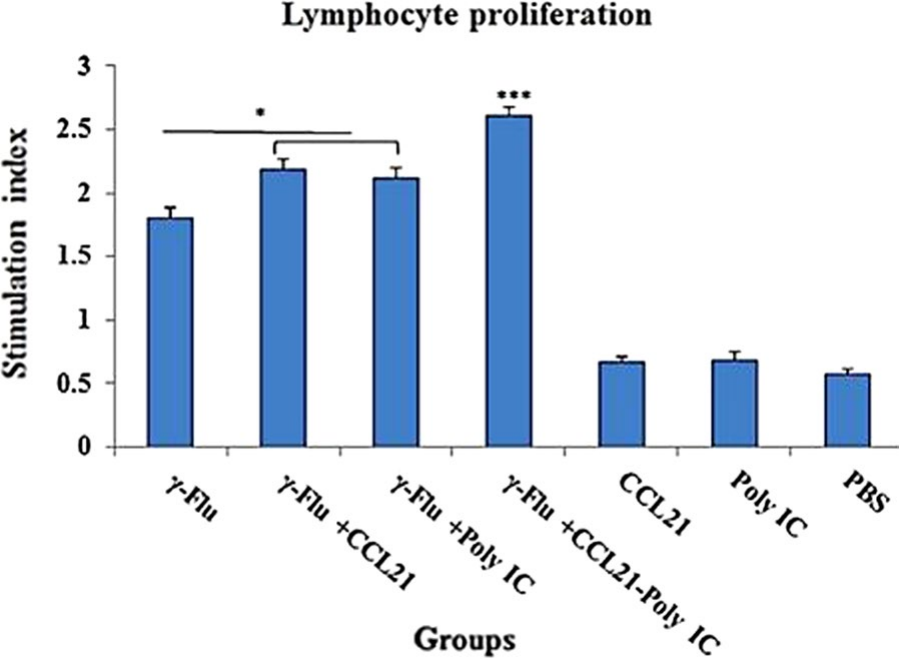
lymphoproliferative activity of splenocytes after in vitro stimulation with γ-PR8 (4 μg/ml) as the specific antigen. One week following the third immunization, the supernatants from restimulated spleen cells *were collected and then subjected to* lymphocyte proliferation analysis using the MTT assay. Values are presented as mean ± SD of three tested mice in each group. The levels of significance are determined by One way ANOVA and indicated by stars as follows: * p ˂ 0.05, *** p ˂ 0.001. Cell proliferation of the γ-Flu*-CCL21-Poly (I:C)* group was significantly higher than those in other vaccinated and control groups (p < 0.001)

*To determine whether CCL21 and Poly (I:C) influenced cellular immune responses,* the lymphoproliferative activity of spleen cells *following *in vitro restimulation with inactivated influenza virus was evaluated one week after *the last immunization. As demonstrated in *Fig. 4*, for* splenocytes *from* γ-Flu*-CCL21-Poly (I:C) vaccinated mice, the treatment induced significant proliferation compared with the other groups* (p < 0.001), *whereas* γ-Flu*-CCL21 and* γ-Flu*-Poly (I:C) groups exhibited similar levels of T-cell proliferation response. However, for* these mice, the level of lymphocyte proliferation *was higher than those primed with the* γ-Flu *alone* (p < 0.05)*, indicating the trend of vaccine adjuvants in* increasing the magnitude of *cell proliferation*. *As expected, spleen cells of mice in control groups including CCL21, Poly (I:C), and PBS showed no significant elevation of lymphoproliferative activity.*

### CCL21-Poly (I:C) as a combination adjuvant improved cellular immune responses to the γ-Flu vaccine

**Fig. 5:**
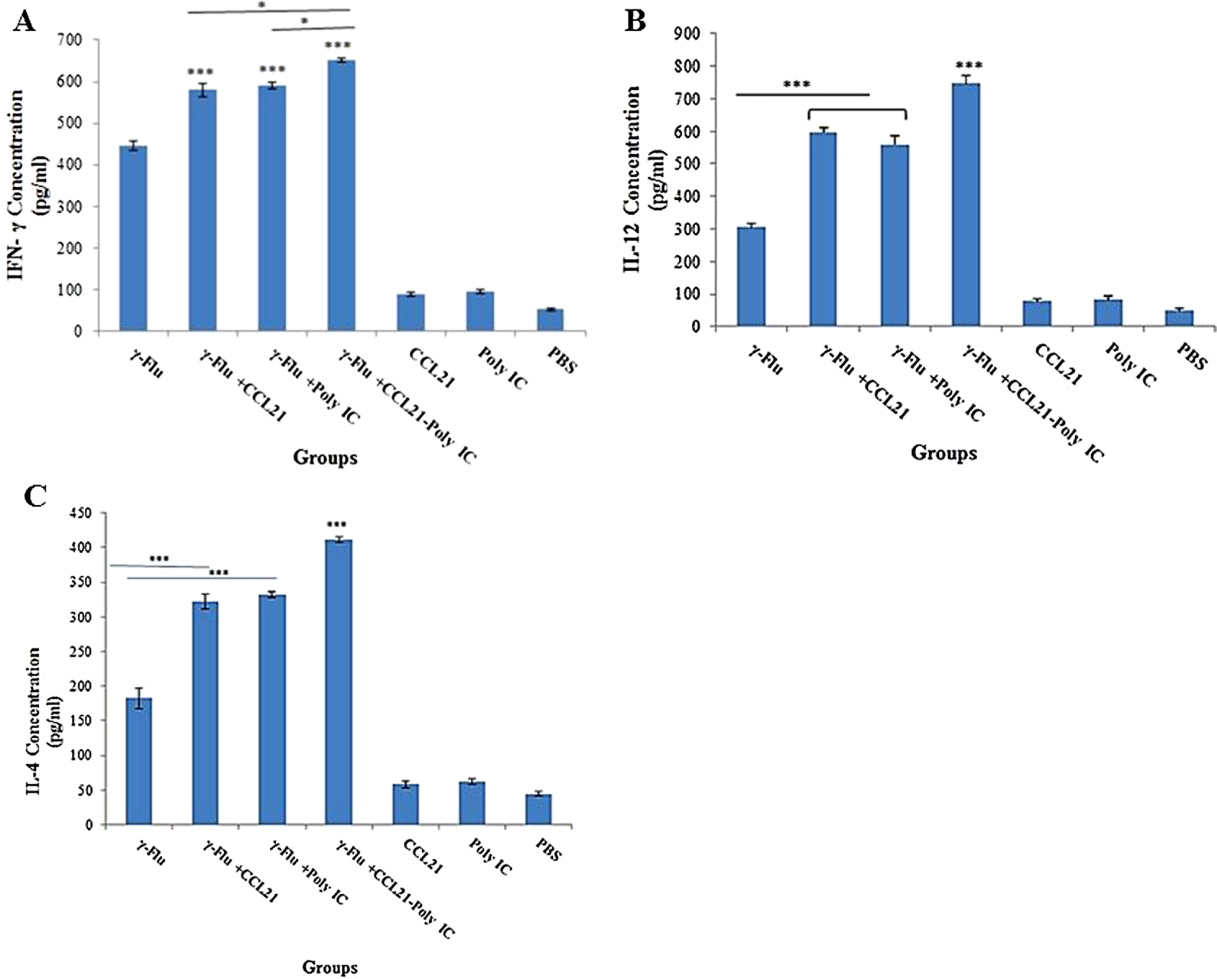
Th1 (IFN-γ (**a**), IL-12 (**b**)) and Th2 (IL-4 (**c**)) cytokine profiles in the splenocyte culture supernatants restimulated with gamma-inactivated influenza virus. Results are representative of three independent experiments that are expressed as the mean ± SD. ***indicates a statistically significant difference between the γ-Flu*-CCL21-Poly (I:C)* group with that of the γ-Flu group, as determined by One way ANOVA (p < 0.001). The graphs also show a statistically significant difference between all vaccinated and control groups (p < 0.001)

To further evaluate the type of immune response developed in immunized mice, on day 21, *the expression rate of* IFN-γ and IL-12*, as* indicators for Th1 *response, as well as the concentration of IL-4, as a hallmark for the Th2 biased responses, in* the supernatants from restimulated spleen cells *was tested. The results of the ELISA assay indicated that the level of Th1 and Th2 cytokines in all vaccinated groups was significantly higher compared to control groups* (p < 0.001)*.* As seen in Fig. 5*,* a trend toward significantly higher expression level of IFN-γ, IL-12*, and IL-4* was noted in mice given γ-Flu *supplemented with any adjuvant alone* or in combination with each other*,* as compared with those receiving γ-Flu, *CCL21, Poly (I:C), and PBS* (p < 0.001). Also, the concentration of IFN-γ in splenocyte culture supernatants were detected at a greater level in mice vaccinated with γ-Flu*-CCL21-Poly (I:C) compared to mice given* γ-Flu *plus CCL21 or Poly (I:C)* (p < 0.05) (Fig. 5a). A similar trend as for the concentration of IFN-γ was also observed for IL-12 and IL-4 in splenocyte culture supernatants (p < 0.001) (Fig. 5b, c)*. Th1- and Th2-cytokine-secreting lymphocytes up-regulated considerably in the spleen of mice treated with* γ-Flu*-CCL21 and* γ-Flu*-Poly (I:C) than those given* γ-Flu *alone* (p < 0.001)*. However,* the level of IL-12 was approximately equivalent in the mice that received *the* γ-Flu with each adjuvant separately*. Importantly, all* adjuvanted vaccines (notably γ-Flu*-CCL21-Poly (I:C) platform)* afforded higher levels of IFN-γ and IL-12 expression than that of IL-4, indicating that vaccine adjuvant incorporation strategy could elicit more potent Th1 and Th2 balanced responses, with more biased toward Th1, to γ-inactivated influenza vaccine. *No significant difference in the concentration of* IFN-γ, IL-12*, and IL-4 was observed in the control groups.*

### Combining CCL21 with poly (I:C) as adjuvant offered a more potent cytotoxic immune response against the γ-Flu vaccine

**Fig. 6:**
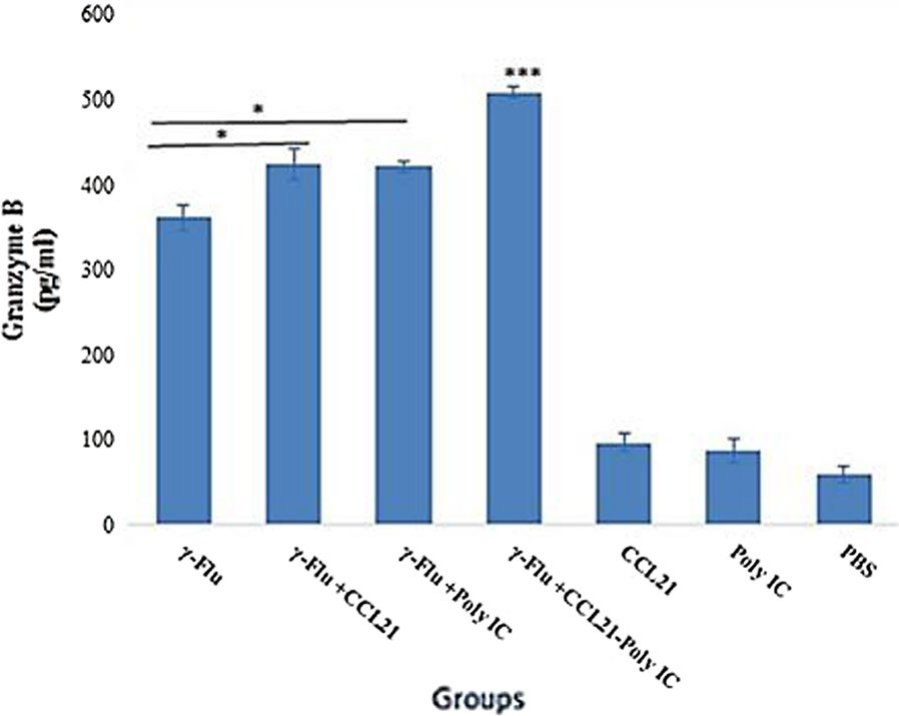
Cytotoxic activity in the vaccinated and control groups. The highest concentration of granzyme B was found in cell supernatants of mice with γ-Flu*-CCL21-Poly (I:C)* regime and a statistically significant difference (p < 0.001) was also observed between vaccinated and control groups. All results are reported as the mean ± SD (n = 3). *displays significant difference compared with mice in the γ-Flu group, as analyzed by One way ANOVA (p < 0.05)

*Since cytotoxic lymphocytes play a crucial role in host defense against influenza infections, the protein content of cytotoxic lymphocyte-derived Granzyme B* in splenocyte culture supernatants of *immunized mice was measured using a quantitative sandwich ELISA assay on day 21. As revealed in *Fig. 6*,* the vaccine-induced cytotoxicity responses were much higher than the responses induced by control groups (p < 0.001). *Furthermore,* the vaccine formulation containing the *CCL21 and the Poly (I:C)* as adjuvant significantly increased *GrB protein levels* compared to all other vaccinated groups (p < 0.001), indicating a medically significant impact of *CCL21 and Poly (I:C)* co-administration on the γ-Flu-induced lymphocyte-mediated cytotoxicity. The rate of *GrB* expression in splenocytes from mice given *Poly (I:C) or CCL21 adjuvanted* vaccine was also higher than that elicited in spleen cells from the γ-Flu group (p < 0.05), representing the adjuvanticity of *CCL21 and Poly (I:C)* when added separately to the inactivated vaccine. *There was no significant difference between the groups that received CCL21, Poly (I:C), and PBS in inducing* cytotoxic *lymphocytes to release granzyme B.*

### The CCL21-Poly (I:C) combination adjuvant significantly reduced inflammatory mediators levels (notably IL-10) in lung homogenate samples

**Fig. 7:**
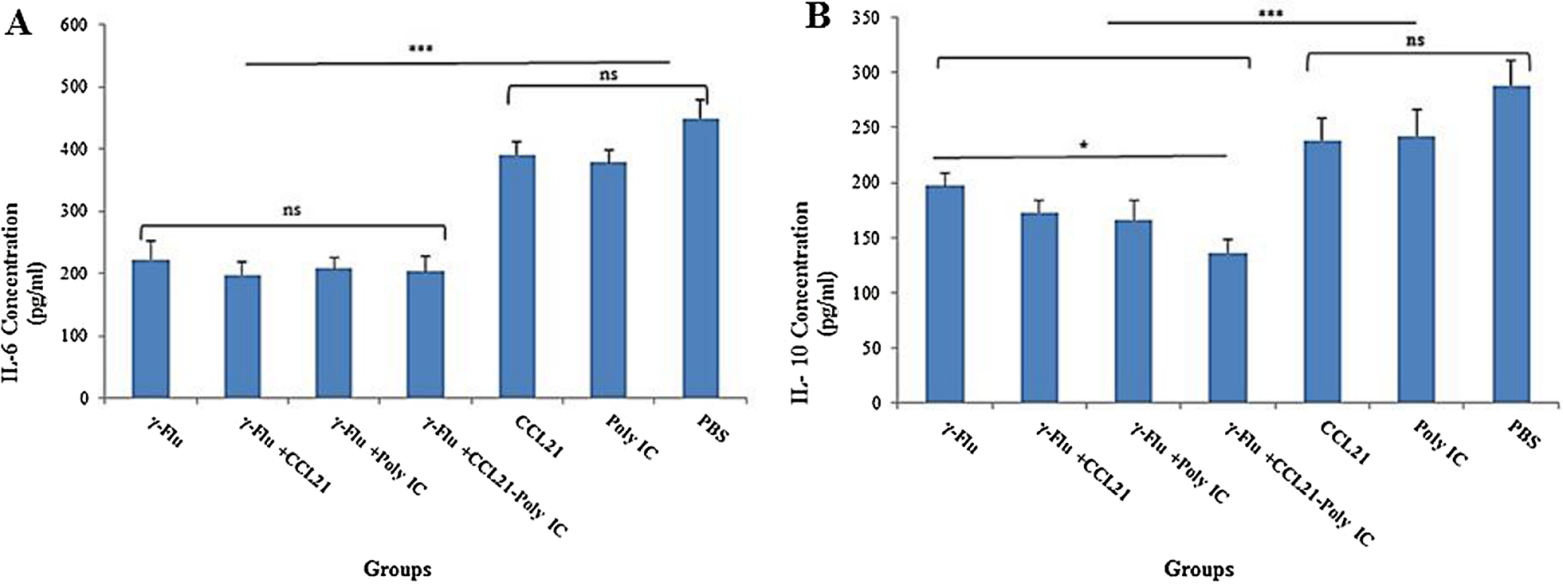
Vaccine-associated inflammatory response in lung homogenates. Data are expressed as the mean ± SD (n = 3) and statistical analysis was conducted by one-way ANOVA. Among different vaccine candidates, a statistically significant difference (p < 0.05) in IL-10 values for the γ-Flu*-CCL21-Poly (I:C)* group was detected. However, no significant difference in IL-6 levels was observed between vaccinated groups**.** *** indicates a statistically significant difference between the treated groups (p < 0.001) compared with control groups (CCL21, Poly (I:C), and PBS)

*To study whether combining CCL21 and Poly (I:C) as mucosal adjuvant with the* γ-Flu *vaccine has the potential to reduce the number of cells secreting inflammatory cytokines, the concentration of IL-6 and IL-10 from* lung homogenates of immunized mice *was measured using the ELISA method after one week of the third immunization. As shown in *Fig. 7*, compared to the three control groups, both IL-6 and IL-10 cytokines were significantly decreased when mice received* γ-Flu *alone or* with any of the three adjuvanted groups (p < 0.001)*. However, there was no difference in numbers of IL-6-secreting cells between the groups vaccinated with* γ-Flu, γ-Flu*-CCL21,* γ-Flu*-Poly (I:C), and* γ-Flu*-CCL21-Poly (I:C) *(Fig. 7a). The results also illustrated that the level of IL-10 was approximately equivalent in the mice that given *the* γ-Flu with *or without* each adjuvant separately *and only mice immunized with the* γ-Flu*-CCL21-Poly (I:C) had lower IL-10-secreting cells than mice given* γ-Flu *alone* (p < 0.05) (Fig. 7b). Between the three control groups, there was no significant difference in IL-6 and IL-10 levels. *Thus, any adjuvant* alone could be equally effective in modulating inflammatory responses of γ-inactivated influenza vaccine*, but only the co-administration of CCL21 and Poly (I:C) significantly reduced* the numbers of IL-10-producing cells in the lung after lethal viral challenge.

### Intranasal administration of γ-Flu vaccine accompanied by the CCL21-Poly (I:C) combination adjuvant provided superior protection to lethal influenza virus challenge and reduced lung viral loads

**Fig. 8:**
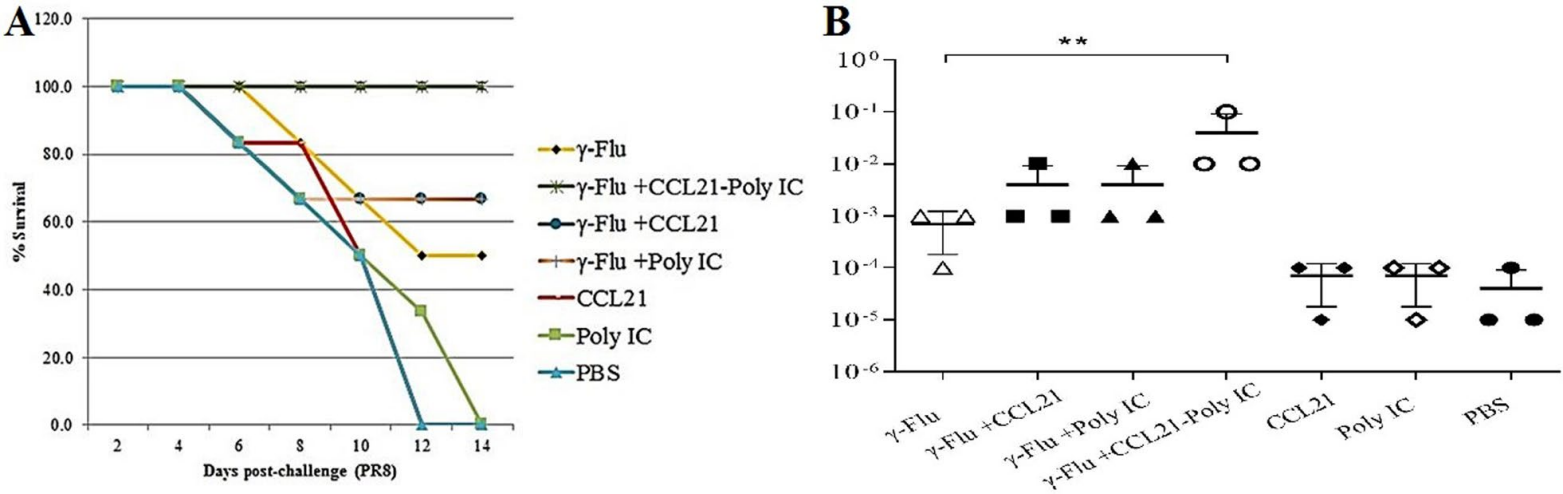
Intranasal vaccination with γ-Flu*-CCL21-Poly (I:C) elevates* protective responses at a high viral challenge dose. One week following the third immunization, mice were infected intranasally with 10 LD50 of A/PR/8/34 (H1N1) and monitored for 14 days to determine survival changes. A loss of 25% of total body weight was used as an endpoint. Survival data are presented as the percentages of animals surviving among the total number monitored (**a**). On day 4 post-challenge, three mice in each group were sacrificed, and lung viral loads were determined by MDCK/HA assay (**b**). ** Indicates statistically significant difference between the with γ-Flu-CCL21-Poly (I:C) and γ-Flu group (p < 0.01), as calculated by one-way ANOVA. The graph also shows a statistically significant difference between all vaccinated and control groups (p < 0.001) (**b**)

*The challenged mice with* 10 LD50 of A/PR/8/34 (H1N1) influenza virus *were monitored daily for 14 days to assess whether the improved antigen-specific immune responses detected in adjuvanted groups could protect animals against* high-dose challenge. *As expected, none of the mice in the CCL21, Poly (I:C), and PBS groups survived; all died* between days *12 and 14 post-infection* (Fig. 8a). In contrast, immunization with the γ-Flu vaccine alone provided 50% protection*, much lower than* γ-Flu-*CCL21 and* γ-Flu*-Poly (I:C)* (p < 0.05)*. As observed in *Fig. 8,* although the co-administration of CCL21 or Poly (I:C) individually with the* γ-Flu vaccine elevated *survival rates*, no statistically significant difference based on the type of adjuvant was found*. Notably,* *100*% vaccine-induced protection *was provided when mice received* γ-Flu *plus the CCL21-Poly (I:C) combination adjuvant.*

We also carried out further assay to determine lung virus loads by determination of viral titers in lung lysate on MDCK by a hemagglutination assay at day 4 after challenge. As shown in Fig. 8b, lung viral loads were significantly decreased when mice received γ-Flu alone or with any adjuvants, compared to the three control groups (p < 0.001). the results also showed that virus titers in the lung were reduced in γ-Flu plus the CCL21-Poly (I:C) group, while the viral loads were less than when the CCL21 and Poly (I:C) adjuvant were administered individually, but no statistically significant difference was found. However, vaccinated mice with γ-Flu plus the CCL21-Poly (I:C) showed significantly lower lung viral loads than γ-Flu alone (p < 0.01). The results indicate that there is a close correlation between the use of vaccine adjuvant formulations and more effective protection against the lethal challenge of the homotypic influenza virus*.*

## Discussion

Vaccination has been developing as the most assured strategy of reducing the problems associated with antiviral therapy in patients contending with the influenza virus, particularly of reducing medical expenses [30]***. ***However, the efficacy of current vaccine platforms can be limited by (a) the high prevalence of antigenic variations in the influenza viruses; (b) the lack of cross-protective immunity [12, 31]; (c) poor immunogenicity among people with risk factors for infection; and (d) vaccine shortage at pandemic events due to their lengthy production time [12, 32, 33]. Accordingly, the genesis of a novel, more sophisticated generation of vaccines should be considered [12].

Preserving the most of immunostimulatory pathogen's epitopes upon gamma-inactivation, which are needed for the induction of an effective coverage against heterologous strains, the high penetration power of γ-rays into biological materials, high safety profile due to impossibility of returning γ-inactivated viruses to virulent type, low requirement for staff training, and the feasibility of rapid preparation of vaccine at large-scale, have led to increased global-attention to gamma-rays as an attractive approach for vaccine manufacturing [1, 34].

Compared to other forms of inactivation approaches, particularly chemical and UV treatments, and regarding the ability of gamma radiation to inactivate pathogens at the deeply frozen state and subsequently to reduce the oxidative alterations of protein epitopes by minimizing free radicals damage provided through water radiolysis, the antiviral activity of ionizing radiation is mainly attributed to preferential absorbance of the gamma photons by nucleic acids, leading to loss of genome integrity**.** Therefore, efficient cellular uptake and un-coating of inactivated Flu viruses is the most probable mechanism by which gamma radiation can induce cross-reactive cytotoxic T- lymphocyte (CTL) responses [5, 34]. Recent studies in mice with gamma-inactivated influenza whole-virion vaccine have revealed its superior efficacy in improving antibody-mediated immunity and inducing the high-level of cellular immune responses, as well as in conferring the homologous or *heterologous immunity* [1–3, 6]. We report here that the mouse-adapted human influenza virus strain A/PR/8/34 [A/Puerto Rico/8/34 (H1N1)], rendered non-infectious by exposure to 28 kGy of gamma-irradiation, can: (I) induce significant virus-specific humoral, mucosal, and cell-mediated immunity in mice; (II) modulate the lung inflammatory responses; and most importantly (III) protect against lethal infection by homotypic influenza viruses, which is consistent with previously published work.

It is important to note that due to the lack of precise optimization of radiation conditions coupled with the Bremsstrahlung process, there is typically the possibility of introducing unwanted alterations in vaccine epitopes, leading to reduction in the effectiveness of the gamma-inactivated vaccine [6, 7]. Furthermore, upon the early attempts for the annual influenza vaccination and due to the whole inactivated influenza vaccine adverse events, safer vaccines (i.e., the split virion influenza vaccine) were developed [12]. Besides, nasal vaccines, notably those that influence the quality and quantity of the adaptive immunity and subsequently induce a robust protective T-cell immune response, are composed of antigens and adjuvants [8–10]. Thus, it seems that the nasal gamma-based inactivated Flu vaccines require adjuvants to reduce their reactogenicity, as well as to improve their immunogenicity. We have previously discussed the superiority of plasmid encoding mouse interleukin-28B (mIL-28B) as a mucosal adjuvant in terms of improved immunity to the whole gamma-irradiated influenza A (subtype H1N1) vaccine [11]. However, there is an increasing demand for safer and more effective vaccine formulations to decline annual influenza fatality rate.

Double-stranded RNA analog Poly (I:C) contributes to the up-regulation of multiple signaling pathways, which leads to increase in DC presentation capacity and in DC migration to the lymphatic organ where the adaptive immunity is shaped [13]. The influence of intranasal administration of Poly (I:C) as a mucosal adjuvant with the vaccine candidate to facilitate the elicitation of a strong adaptive immunity has been well documented [8, 23, 24]. Ichinohe et al*.* demonstrated that intranasal co-delivery of poly (I:C) as an adjuvant could elevate the level of alpha/beta interferons, as well as Th1- and Th2-associated cytokines induced by the inactivated influenza virus HA vaccine [14]. Another study conducted by Zang et al. revealed the efficacy of poly (I:C) as a mucosal adjuvant in elevating both humoral and cellular immune responses to inactivated H9N2 vaccine when included in the vaccination regimen [10]. On the other hand, poly (I:C), administered intranasally in aged mice, has recently been shown to induce the protection against lethal respiratory virus infections, without any side effects, by up-regulating the expression of IFN-β, IFN-γ, IL-1β, and tumor necrosis factor (TNF) in the lungs, indicating the potential applicability of poly (I:C) as a prophylactic agent in aged individuals with risk factors for severe acute respiratory syndrome coronavirus (SARS-CoV) or influenza A virus (IAV) infections [35].

The immunotherapeutic capability of CCL21, as a CCR7 ligand, has also been underscored by its ability to induce the CCR7-dependent recruitment of immunocytes into the secondary lymphoid tissues, resulting in the development of adaptive immunity (notably T-cell proliferation) [15, 19, 20]. The efficacy of CCL21 as a mucosal adjuvant to enhance the specific types of immunity was evaluated by Toka and colleagues. The results showed that intranasal vaccination of mice with a plasmid DNA or recombinant vaccinia virus encoding herpes simplex virus gB accompanied by the CCL21 adjuvant greatly improved the production of IgG and IgA in the serum and vaginal samples, respectively, and highly increased cytotoxic T lymphocyte responses. Furthermore, due to a rapid recall response, CCL21 adjuvanted vaccine provided the significant protection against the viral challenge [26]. Hence, the objective of the present study was to apply the murine recombinant CCL21 and poly (I:C), either alone or in combination with each other, as mucosal adjuvants to augment the immunogenicity of the gamma-irradiated H1N1 vaccine.

The results illustrated that compared to the γ-Flu alone immunized group, intranasal co-administration of CCL21or poly (I:C) individually with the γ-Flu vaccine induced a strong specific immune response, as evidenced by increasing the levels of Th1-type cytokines including IFN-γ and IL-12 compared to the concentration of IL-4 as the Th2 indicator, along with an increase in the ratio of the Th1-associated IgG2a to the Th2-associated IgG1 subtype. Increased serum IgG responses to vaccine candidate, as well as a higher trend toward the proliferative response and the release of granzyme B in the spleen of mice treated with the γ-Flu plus CCL21 or poly (I:C), as compared with those receiving γ-Flu *alone*, was correlated with more effective protection after a lethal influenza virus challenge (10 LD50). Furthermore, both CCL21 and Poly (I:C) adjuvants could prompt higher rates of IgA response in BAL samples compared with the non-adjuvanted vaccine. Nonetheless, based on the ELISA data, the level of antigen-specific mucosal *IgA* antibody in the nose was similar between the mice received *the* γ-Flu *alone or* with each adjuvant separately. However, our results also showed that the IgA response in the upper (nasal washes) and the lower (BAL fluids) respiratory tract *was significantly higher than those primed with CCL21, poly (I:C), and PBS*. It should be noted that mucosal immunity represents the host's first line of defense against influenza virus, which is involved in preventing the spread of infectious particles by IgA-mediated virus neutralization [36, 37]. Upper and lower respiratory tract sampling to assess the mucosal IgA concentrations showed that the level of IgA in the BAL fluids was higher than IgA titers in nasal washes. This finding is consistent with a study conducted by Song and his group [38] and our previously published work [11], reinforcing the key role of the presence of antigen-specific neutralizing antibodies in the lower respiratory tract in preventing influenza virus infection. Taken together, the current study highlighted the adjuvanticity of CCL21 or poly (I:C) to increase the immunogenicity of the γ-Flu vaccine, when co-delivered to the mucosal sites.

*It is important to note* that upon combining CCL21 with poly (I:C), a more potent cell- and antibody-mediated immunity in response to the γ-Flu vaccine was elicited, resulting in 100% survival following a high lethal dose challenge of A/PR/8/34 (H1N1) influenza virus. *The* CCL21 and poly (I:C) combination adjuvant was also able to strongly promote Th1-oriented responses with a higher ratio of IgG2a/Th1 to IgG1/Th2, as well as with an increased Th1-related cytokines (IFN-γ and IL-12). A comparison of the three adjuvant combinations revealed that CCL21 in conjugation with poly (I:C) effectively improved the mucosal antibody response in the BALF and nasal samples, as well as significantly promoted the vaccine-induced cytotoxic responses by increasing the levels of granzyme B and the CD4^+^ T-cell proliferation. Consequently, intranasal treatment of mice with CCL21 and Poly (I:C) combination adjuvant was more effective than any adjuvant alone, indicating the synergistic effects of CCL21 and poly (I:C) on the vaccine-induced immune responses.

Next, we analyzed the level of IL-6- and IL-10 cytokines in the lung homogenates of immunized mice to assess the effect of CCL21 and poly (I:C), either alone or in combination with each other, as mucosal adjuvants on the modulation of inflammatory responses. New data highlight the correlation between the excessive production of IL-6, as a major inflammatory mediator, and the *hyperinflammatory* state during influenza infection and subsequent increase in the illness severity [39–41]. IL-10 is also essential for dampening the magnitude of antigen-specific immune responses to reduce host damage by negatively regulating innate and adaptive immunity. However, the exact role of IL-10 in influenza pathogenesis remains unclear and controversial results (detrimental or protective roles) for IL-10 upon viral infection have been reported [42–44]. Recent studies in mice have shown that IL-10 levels are inversely related to the severity of influenza A virus-induced disease. Sung and his group revealed the adverse effect of IL-10 on protective pulmonary antibody immunity by interfering with the initial CD4^+^ T-cell function. A detrimental effect of IL-10 on the development of Th17-type immune responses and subsequent reduction in survival rate following a lethal challenge with a high dose of influenza has also been reported recently [42, 43]. Our findings demonstrate that the γ-Flu vaccine alone or combined with CCL21, poly (I:C), or CCL21-poly (I:C) efficiently reduced IL-6 concentrations, but differences were not significant. A similar trend was also observed for IL-10 in lung homogenates, with less IL-10 secretion obtained from immunized mice with the γ-Flu *alone or with* each adjuvant separately. However, the ability of CCL21 and poly (I:C) combination adjuvant in conjunction with the γ-Flu vaccine to attenuate the production of IL-10 is highly desirable, which provides further support for the concomitant administration of CCL21 and poly (I:C) as a potent mucosal adjuvant.

## Conclusion

The current study illustrates the importance of incorporating CCL21 and Poly (I:C) combination adjuvant *in* the γ-Flu vaccine formulation *for* improving vaccine-mediated protection against intranasal challenge with 10 LD50 of H1N1 influenza viruses *by increasing the ratio of IgG2a, IFN-* γ, and IL-12 as Th1 indicators and the level of IgA- and IgG-manifested *humoral immunity*, as well as by increasing lymphocytic proliferation and cytotoxic activity of spleen cells. Furthermore, the CCL21 and Poly (I:C) combination adjuvant has a potent propensity to reduce inflammatory cytokines, notably IL-10, indicating the synergistic effects of two different types of immunological adjuvants on vaccine responses.

## Data Availability

The datasets used and analysed during the current study are available from the corresponding author on reasonable request.

## Abbreviations

γ-Flu: Gamma-irradiated influenza A virus
DC: Dendritic cell
Poly (I:C): Polyriboinosinic-polyribocytidylic acid
CCL21: Chemokine (C–C motif) ligand 21
dsRNA: Double-stranded RNA
TLR3: Toll-like receptor 3
MDA5: Melanocyte differentiation-associated 5
RIG-I: Retinoic acid-inducible gene I
*Nlrp3*: NLR *pyrin domain* containing *3*
IFN-1: Type 1 interferon
CCR7: CC chemokine receptor 7
Th1: T-helper 1
HSV-1: Herpes simplex virus type 1
MDCK: Madin-Darby Canine Kidney
MOI: Multiplicity of infection
CPEs: Cytopathic effects
TCID50: Median tissue culture infectious dose
LD50: Median lethal dose
HA: Hemagglutination assay
SDS-PAGE: Sodium dodecylsulfate-polyacrylamide gel electrophoresis
ELISA: Enzyme-linked immunosorbent assay
RT: Room temperature
HRP: Horseradish peroxidase
TMB: Tetramethylbenzidine
OD: Optical density
BAL: Bronchoalveolar Lavage
SI: Stimulating index
*GrB*: Granzyme B
CTL: Cytotoxic T- lymphocyte
mIL-28B: Mouse interleukin-28B
TNF: Tumor necrosis factor
SARS-CoV: Severe acute respiratory syndrome coronavirus
IAV: Influenza A virus
