# Correction to: A formulated poly (I:C)/CCL21 as an effective mucosal adjuvant for gamma‑irradiated influenza vaccine

**DOI:** 10.1186/s12985-021-01684-z

**Published:** 2021-11-02

**Authors:** Ailar Sabbaghi, Masoud Malek, Sara Abdolahi, Seyed Mohammad Miri, Leila Alizadeh, Mehdi Samadi, Seyed Reza Mohebbi, Amir Ghaemi

**Affiliations:** 1 Department of Influenza and Other Respiratory Viruses, Pasteur Institute of Iran, P.O.Box: 1316943551, Tehran, Iran; 2 Department of Microbiology, Science and Research Branch, Islamic Azad University, Tehran, Iran; 3 Shefa Neuroscience Research Center, Khatam Alanbia Hospital, Tehran, Iran; 4 Department of Medical Virology, Tehran University of Medical Sciences, Tehran, Iran; 5 Gastroenterology and Liver Diseases Research Center, Research Institute for Gastroenterology and Liver Diseases, Shahid Beheshti University of Medical Sciences, Tehran, Iran

## Correction to: Virol J (2021) 18:201 10.1186/s12985-021-01672-3

Following publication of the original article [1], we have been informed that author Seyed Reza Mohebbi has been incorrectly affiliated.

The author affiliation has been updated above and the original article [1] has been corrected.
